# Discrimination of three types of homopolymers in single-stranded DNA with solid-state nanopores through external control of the DNA motion

**DOI:** 10.1038/s41598-017-08290-6

**Published:** 2017-08-22

**Authors:** Rena Akahori, Itaru Yanagi, Yusuke Goto, Kunio Harada, Takahide Yokoi, Ken-ichi Takeda

**Affiliations:** Hitachi Ltd., Research and Development Group, Center for Technology Innovation – Healthcare, 1-280, Higashi-koigakubo, Kokubunji, Tokyo 185-8601 Japan

## Abstract

To achieve DNA sequencing with solid-state nanopores, the speed of the DNA in the nanopore must be controlled to obtain sequence-specific signals. In this study, we fabricated a nanopore-sensing system equipped with a DNA motion controller. DNA strands were immobilized on a Si probe, and approach of this probe to the nanopore vicinity could be controlled using a piezo actuator and stepper motor. The area of the Si probe was larger than the area of the membrane, which meant that the immobilized DNA could enter the nanopore without the need for the probe to scan to determine the location of the nanopore in the membrane. We demonstrated that a single-stranded DNA could be inserted into and removed from a nanopore in our experimental system. The number of different ionic-current levels observed while DNA remained in the nanopore corresponded to the number of different types of homopolymers in the DNA.

## Introduction

DNA sequencing using nanopores^1–4^ offers the advantage of enabling long-read DNA sequencing without amplification and fluorescence labelling. The four different types of nucleotides in DNA can be identified by measuring the ionic current conducted through a nanopore when DNA passes through the pore. Recently, Oxford Nanopore Technologies, Ltd. began to distribute its biological DNA sequencer (MinION), and several studies of its sequencing capacity have been reported^5–10^. According to one of these reports^5^, MinION achieved single-stranded DNA reads at a level of accuracy greater than 92% accurate. Numerous factors are required to attain such highly accurate nanopore DNA sequencing. From the perspective of sensing techniques, there are two key techniques. One is a fabrication technique that produces a self-assembled nanopore in a biological membrane, with the nanopore having a sufficiently short sensing length to sequence DNA^4^. The other is a technique for controlling DNA motion using a processive enzyme that can ratchet DNA through the nanopore by the advancement of a single-nucleotide unit^4^.

On the other hand, many studies have also been performed to achieve DNA sequencing using solid-state nanopores^11, 12^. Solid-state nanopores are formed using semiconductor-related inorganic materials. Therefore, this approach has an advantage in terms of robustness. Venta *et al*. reported that three types of homopolymers that pass through a silicon nitride nanopore can be distinguished by detecting the change in the ionic current^11^. Moreover, Feng *et al*. reported that four types of homopolymers and monomers can be distinguished using molybdenum disulphide (MoS_2_) nanopores^12^. However, no reports have demonstrated DNA sequencing using solid-state nanopores.

One of the challenges for DNA sequencing with solid-state nanopores is controlling the translocation speed of DNA through a nanopore. When DNA passes through a nanopore via an electric field in ionic solution, the typical dwell time of the DNA in the nanopore is less than 1 μs per nucleotide (μs/nt). This dwell time is too short for the detection of the ionic-current signal derived from each nucleotide when using commercially available amplifiers^13, 14^. Ideally, the dwell time of DNA in a nanopore should be at least 10–1000 μs/nt to enable satisfactory recordings of the signal from each nucleotide.

To reduce the DNA translocation speed through the nanopore, numerous strategies have been proposed^15–26^. For example, Fologea *et al*. demonstrated that DNA translocation speed can be reduced by the addition of ethylene glycol to the ionic solution, and the resulting speed is reduced by as much as 6-fold compared to that observed without ethylene glycol^15^. Kowalczyk *et al*. demonstrated that the DNA translocation speed in a LiCl aqueous solution is reduced approximately 10-fold compared to the speed observed using a KCl aqueous solution^16^. Squires *et al*., Yoshida *et al*., Goto *et al*. and Wang *et al*. used a strategy in which the membrane was coated with various obstacles to decelerate DNA translocation such as: a nanofibre mesh^19^, polyethylene oxide (PEO)-filled nano-cylindrical domains^20^, amine-functionalized beads^21^, and a hydrophilic self-assembled monolayer^22^. Using that approach, the dwell time of the DNA in the nanopore could be increased to approximately 10–100 μs/nt.

Other approaches utilize a DNA-immobilized atomic force microscopy (AFM) probe or bead. Control of the translocation speed of the immobilized DNA through the nanopore is achieved through control of the motion of the probe or the bead using an actuator or optical potential^27–29^. Hyun *et al*. and Nelson *et al*. demonstrated that DNA immobilized on the probe could be inserted into and pulled out from the nanopore using a piezo actuator. The dwell time of the DNA was greater than 100 μs/nt.

In this study, we also used a DNA-immobilized probe with actuators. However, in our system, the probe has a large, flat area on which many single-stranded DNAs (ssDNAs) are immobilized, and this area is larger than that of the nanopore membrane. Therefore, one of the immobilized DNAs can easily enter the nanopore when the flat section of the probe is placed close to the membrane without requiring the probe to scan to determine the location of the nanopore in the membrane. The motion of the probe is controlled using a piezo actuator and stepper motor. Using our system, we measured and analysed the ionic currents observed when various types of ssDNAs were retained in the nanopores. Consequently, we found that the number of the different ionic-current level corresponded to the number of different types of homopolymers in the ssDNA.

## Results

**Figure 1:**
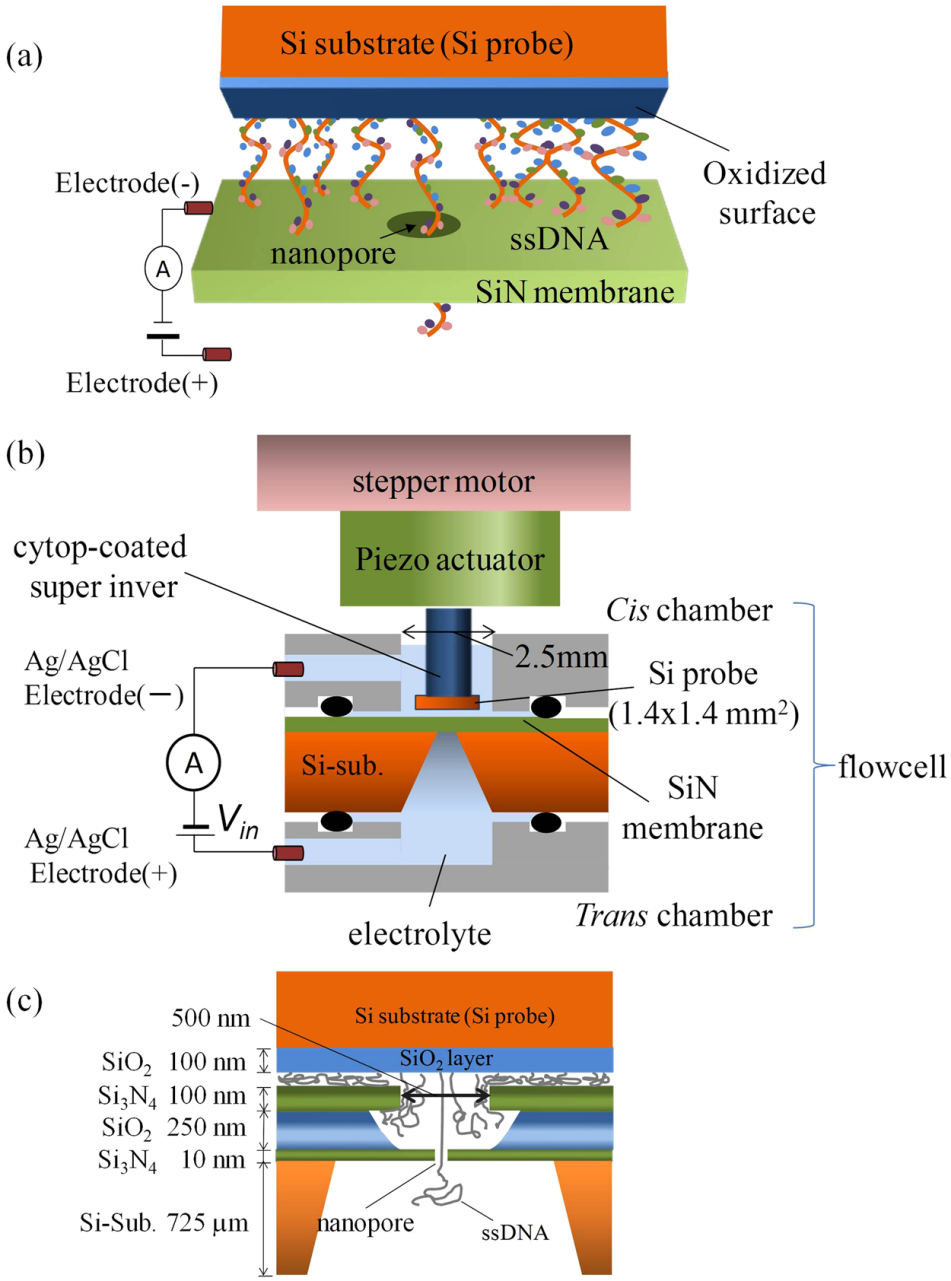
Schematic of the nanopore measurement system. (**a**) Schematic of the ionic-current measurement when DNA that is immobilized on the probe remains in the nanopore. (**b**) Schematic of the measurement setup. (**c**) Close-up schematic for around the nanopore.

Figure 1 provides a schematic of our system. Samples of ssDNAs were immobilized on the oxidized surface of a Si substrate using peptide binding with (3-aminopropyl)triethoxysilane (APTES) and glutaraldehyde (Fig. 1(a)). The Si probe was connected to the vertical position controller, which was composed of a piezo actuator and a stepper motor (Fig. 1(b)). The flow cell was composed of two parts: the *cis* and *trans* chambers. The *cis* chamber included a channel to allow the Si probe to approach the vicinity of the nanopore. The Si probe could be driven by the stepper motor (250 nm/step) or the piezo actuator. Both chambers were filled with 1 M KCl aqueous solution, and a Ag/AgCl electrode was immersed in each aqueous solution. The electrodes were connected to a voltage source and an ammeter. Images of the system and the motions of the probe measured by a laser displacement meter are shown in the Supplementary Information, SI-1 and SI-2. The hysteresis in the movement of the Si probe was suppressed by closed loop control of the actuator. The drift of the probe was approximately 0.486 nm/s. All of the parts of the system were mounted on an active vibration isolation system in an acoustic enclosure to reduce the effects of sound and floor vibrations. The vibration characteristics are shown in the Supplementary Information, SI-3, and the positional fluctuations of the probe and membrane chip are shown in the Supplementary Information, SI-4. The probe and membrane chip oscillated in the range of 1-2 nm, respectively. Then, the oscillational fluctuation of the relative distance between the probe and membrane chip is assumed to be up to 3–4 nm. Consequently, the total vertical positional error between the probe and the nanopore is estimated to be 0.486 nm/s (drift motion) + 3–4 nm (oscillation).

Figure 1(c) presents a close-up schematic for the area around the nanopore. The nanopore was fabricated in a 10-nm-thick Si_3_N_4_ membrane. The membrane was an area approximately 500 × 500 nm^2^ squared, created by etching the SiO/Si_3_N_4_ multilayer (250 nm/100 nm) deposited on the Si_3_N_4_ membrane. The diameter of the nanopore was approximately 2 nm, and the nanopore was made by utilizing the dielectric breakdown of the membrane^30, 31^. When the Si probe approaches the membrane, an ssDNA is directed to the nanopore by the electric field near the nanopore.

**Figure 2:**
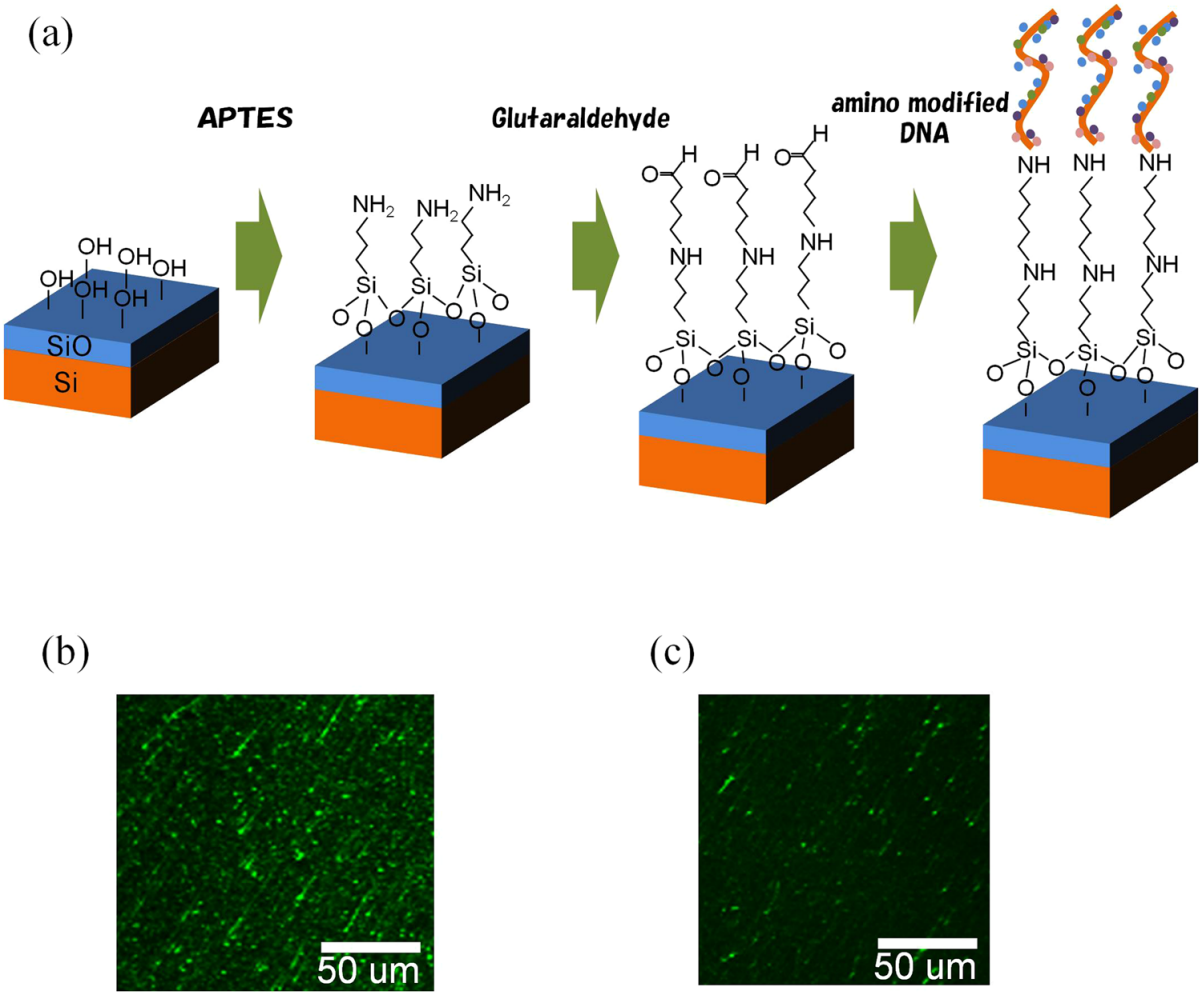
DNA immobilization on the probe. (**a**) Process for immobilizing the DNA on the surface of the probe. (**b**) Fluorescence imaging of the ssDNAs ([(dA)_50_-(dC)_50_]_m_) on the surface of the Si probe when the ssDNAs were bound with glutaraldehyde by peptide coupling. (**c**) Fluorescence image of the ssDNAs ([(dA)_50_-(dC)_50_]_m_) on the surface of the Si probe when the ssDNAs were not bound with glutaraldehyde by peptide coupling.

The process of immobilizing ssDNAs on the probe is illustrated in Fig. 2(a). After the Si surface was oxidized to a thickness of 100 nm, a layer of APTES was formed on the oxidized surface. Then the probe was immersed in a glutaraldehyde solution. Finally, the ssDNAs were bound to the probe with glutaraldehyde by peptide coupling. To confirm that the ssDNAs were bound to the probe, fluorescence observations were performed. The ssDNAs were fluorescently labelled using SYBR® Gold nucleic acid gel stain (Thermo Fisher Science Inc., MA). Figure 2(b) shows the fluorescence observed on the surface of the fabricated probe. Many fluorescent ssDNAs were observed. By contrast, Fig. 2(c) presents an image obtained when the probe was fabricated without the use of APTES and glutaraldehyde. This image confirms that only a few fluorescent ssDNAs bound to the substrate in this context. Additional information about observations of fluorescence is provided in Supplementary Information SI-5.

**Figure 3:**
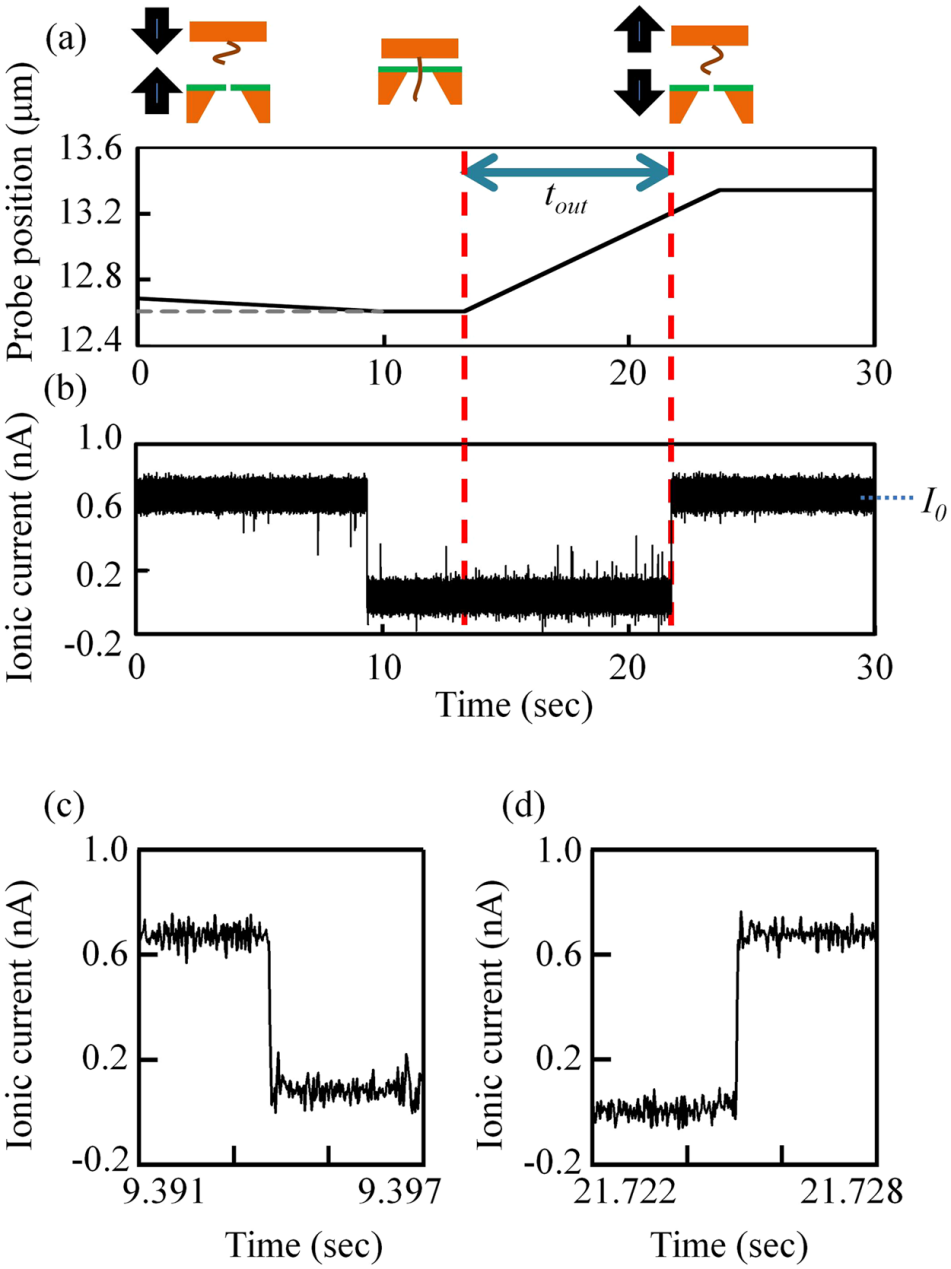
Insertion and removal of the DNA from the nanopore. (**a**) Time trace of the probe position and the (**b**) corresponding ionic current through the nanopore. (**c**) Magnified views around the time points when the ionic current was blocked. (**d**) Magnified views around the time points when the ionic current was recovered.

Using the instrument already described and the DNA-immobilized Si probe, we demonstrated the feasibility of inserting ssDNA into the nanopore and pulling it out of the nanopore using the vertical position controller. Figure 3(a) presents a time trace of the Si probe displacement. In this experiment, 5.3-kb ss-poly(dA) was immobilized on the probe. Details of the preparation process used for the 5.3-kb ss-poly(dA) are described in ref. 24, which reported that the variation in the length of ss-poly(dA) was approximately 5.3-kb ± 0.4-kb. In this paper, we hereafter refer to 5.3-kb ± 0.4-kb ss-poly(dA) as poly(dA)_5.3k_. The Si probe was moved close to the nanopore membrane during the first 10 sec. Then the Si probe motion was stopped for the next 3 sec. The Si probe was subsequently moved upwards and away from the nanopore membrane. Figure 3(b) shows the ionic current through the nanopore at 0.1 V during the displacement of the Si probe. The ionic current was blocked when the probe approached the membrane, and the current recovered to the original current value (i.e., the open pore current, *I*_0_) when the probe was moved away from the membrane by a sufficient amount. This behaviour is consistent with the previous results reported by Hyun *et al*. and Nelson *et al*.^28, 29^, who used a DNA-immobilized tip and a nanopore. In addition, we performed a negative control experiment in which ssDNA was not immobilized on the Si probe (see Supplementary Information SI-6). No ionic-current blockades were observed in this negative control experiment, even when the probe touched the membrane chip.

*I*_0_ was approximately 0.68 nA. In this study, the diameter of the nanopore (*ϕ*) was estimated using the following equation^32, 33^:

1 $${I}_{0}={V}_{{\rm{in}}}\sigma {(\frac{4{{h}}_{{\rm{eff}}}}{{\rm{\pi }}{\varphi }^{2}}+\frac{1}{\varphi })}^{-1},$$

in which *V*_in_ is the applied voltage across the nanopore. Here, σ = 0.105 S/cm is the measured conductance of the KCl buffer solution at 22.5 °C, and *h*_eff_ = 3.75 nm is the average effective thickness of the fabricated nanopores^31^. *h*_eff_ was extracted from the relationship between measured *I*_0_ and the diameters of nanopores measured from TEM images (see Supplementary Information SI-7). The variation in *h*_eff_ was 3.75 ± 0.75 nm. The variation in ϕ can also be estimated. For example, *ϕ* is estimated as 2.11 ± 0.18 nm when *I*_0_ = 0.68 nA at 0.1 V. Figure 3(c) and (d) show magnified views around the time points when the ionic current was blocked or recovered.

**Figure 4:**
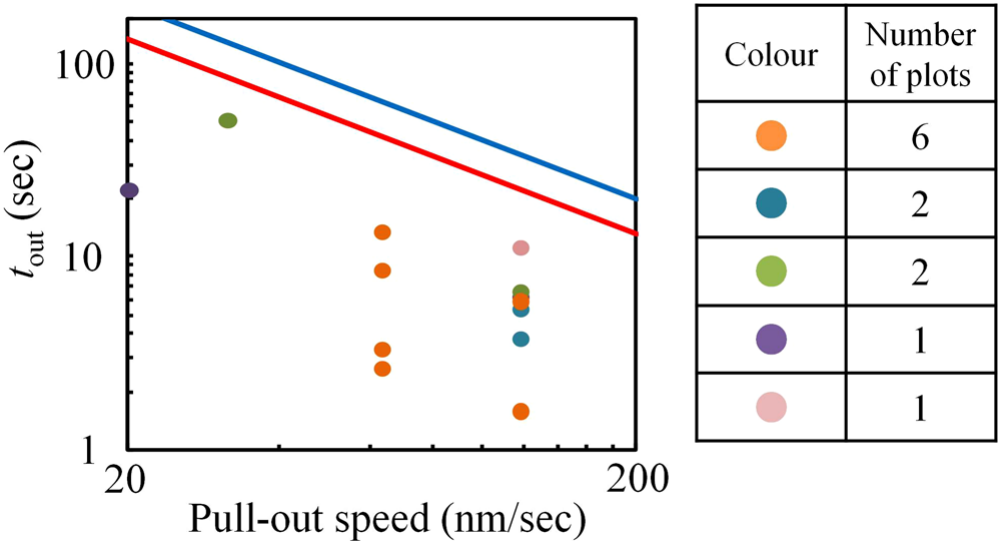
Relationship between *t*_out_ and the pull-out speed of the actuator. A total of 15 experiments were performed and the results have been plotted. The circles plotted present the experimental data. The data acquired using the same nanopore chip are expressed using the same colour for the data points. The red and blue lines represent the calculated limits of *t*_out._

Figure 4 presents the relationship between the time required to pull the poly(dA)_5.3k_ out from the nanopore (*t*_out_), and the pull-out speed of the probe. The circle plots show the experimental data. In this experiment, five different nanopore chips were used. Data acquired using the same nanopore chip is plotted using the same colour. When the same nanopore chip was used for multiple measurements, the n_th_ measurement was performed after pulling poly(dA)_5.3k_ out from the nanopore in the n−1_th_ measurement. For all of the plots in the figure, the approach speed of the probe was set to 7.9 nm/sec and the probe motion was stopped within approximately 1 sec after the ionic current was blocked. The lines represent the theoretical limits of *t*_out_. These values were calculated as: [(length of poly(dA)_5.3k_) − (thickness of SiO_2_/Si_3_N_4_ layer = 350 nm)]/(pull-out speed). The variation (i.e., error estimation) in the theoretical limit of *t*_out_ is expressed as the range between the red line and the blue line. This variation derives from the variation in the length of poly(dA)_5.3k_. As noted above, the variation in the base number of poly(dA)_5.3k_ is 5.3-kb ± 0.4-kb. In addition, according to ref. 34, the variation in the distance between each base of ssDNA in solution was approximately 0.63 ± 0.08 nm. Therefore, the possible variation in the length of poly(dA)_5.3k_ is approximately 2.7 μm (=0.55 nm × 4.9 k) to 4.0 μm (=0.71 nm × 5.7 k). The red/blue line was calculated by setting the length of poly(dA)_5.3k_ at 2.7/4.0 μm. It is reasonable that all of the plots in the figure are under the theoretical limit, which further demonstrates that DNA on the probe can be pulled in and out of the nanopore using our system.

**Figure 5:**
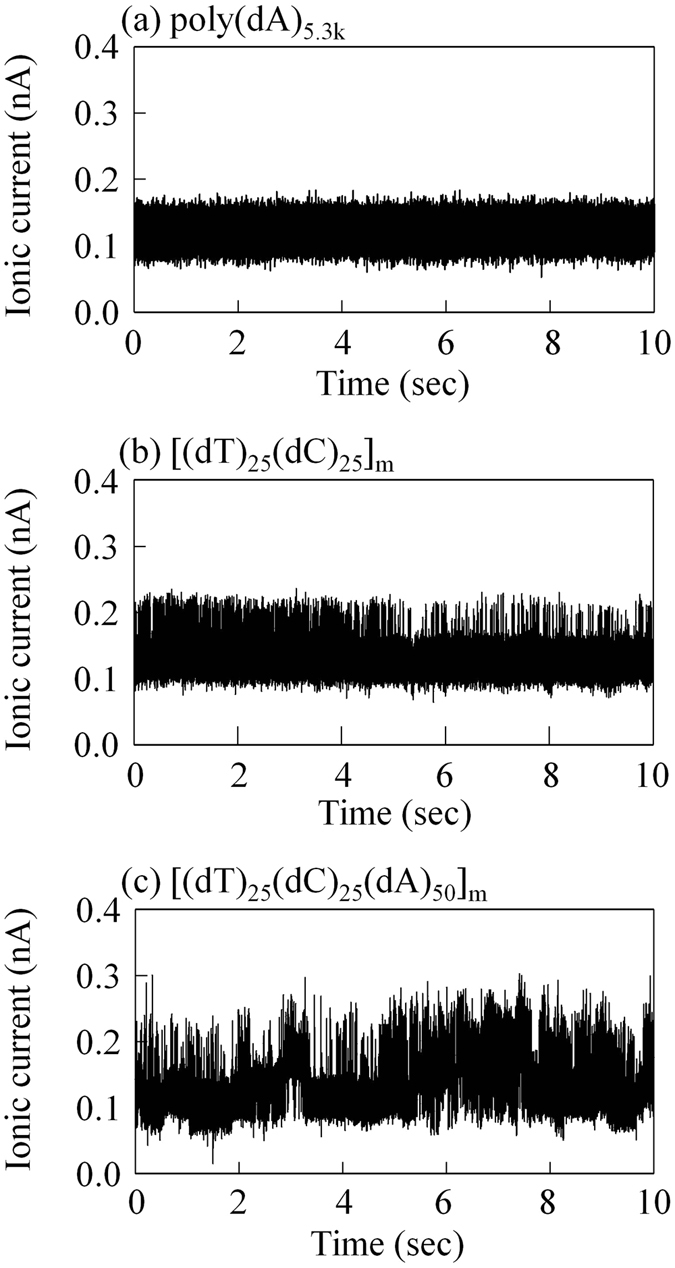
Long-term time trace of the ionic current while the homopolymer or block copolymer remained in the nanopore. A typical time trace of the ionic current for 10 sec while (**a**) poly(dA)_5.3k_, (**b**) [(dT)_25_-(dC)_25_)]_m_, and (**c**) [(dT)_25_-(dC)_25_-(dA)_50_]_m_ remained in the nanopore. Each applied voltage was 100 mV. Each current signal was filtered at 5 kHz. The diameter of each nanopore was (**a**) 2.07 nm, (**b**) 1.93 nm, and (**c**) 2.08 nm.

**Figure 6:**
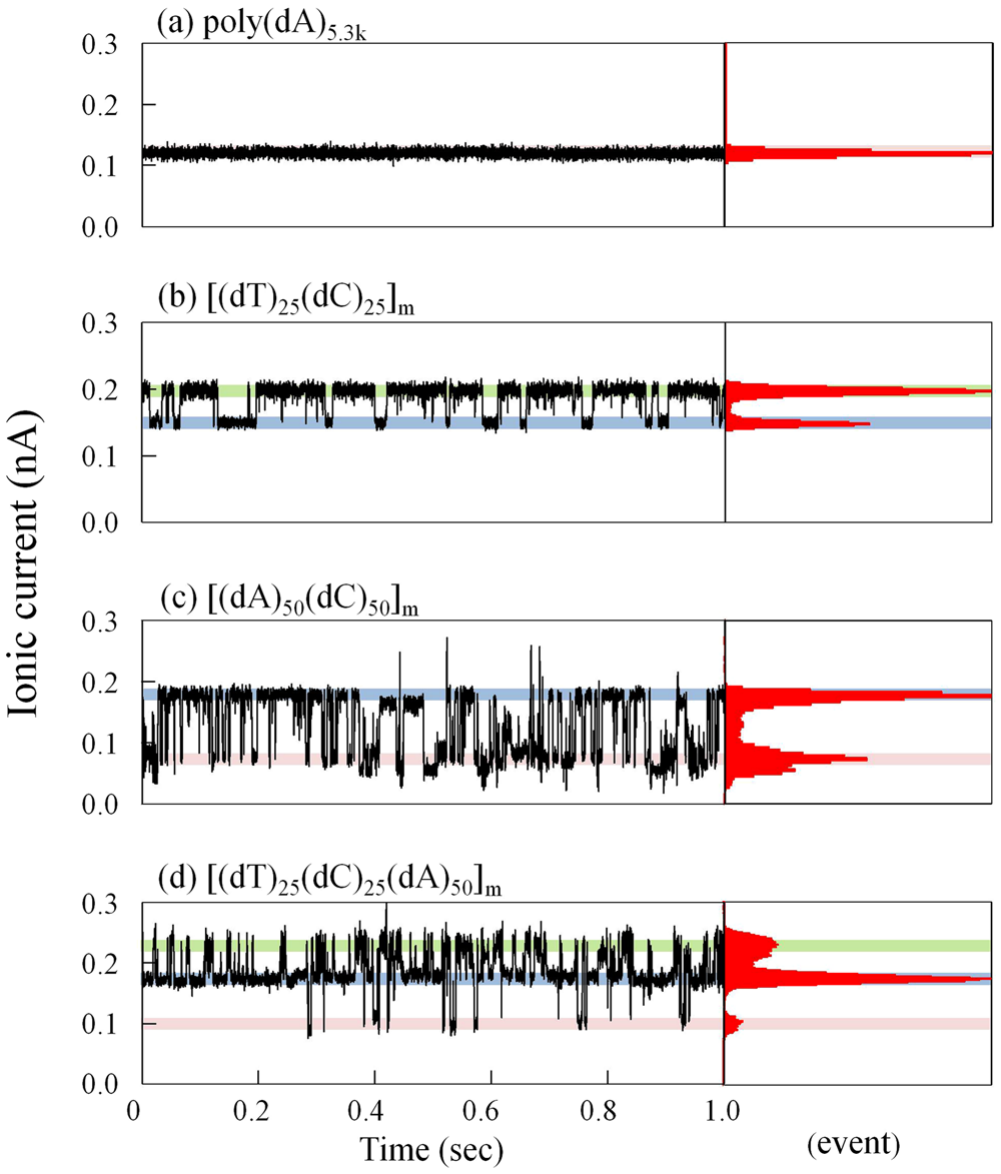
Short-term time trace of the ionic current while the homopolymer or block copolymer remained in the nanopore. Typical time trace of the ionic current for 1 sec while (**a**) poly(dA)_5.3k_, (**b**) [(dT)_25_-(dC)_25_]_m_, (**c**) [(dA)_50_-(dC)_50_]_m_, and (**d**) [(dT)_25_-(dC)_25_-(dA)_50_]_m_ remained in the nanopore. Each applied voltage was 100 mV. Each current signal was filtered at 2 kHz. The values of the ionic-current blockades at the intensity peaks in each histogram were (**a**) 118 pA; (**b**) 148 pA and 197 pA (shaded blue and green, respectively); (**c**) 72 pA and 176 pA (shaded red and blue, respectively); and (**d**) 98 pA, 173 pA, and 222 pA (shaded red, blue and green, respectively).

**Figure 7:**
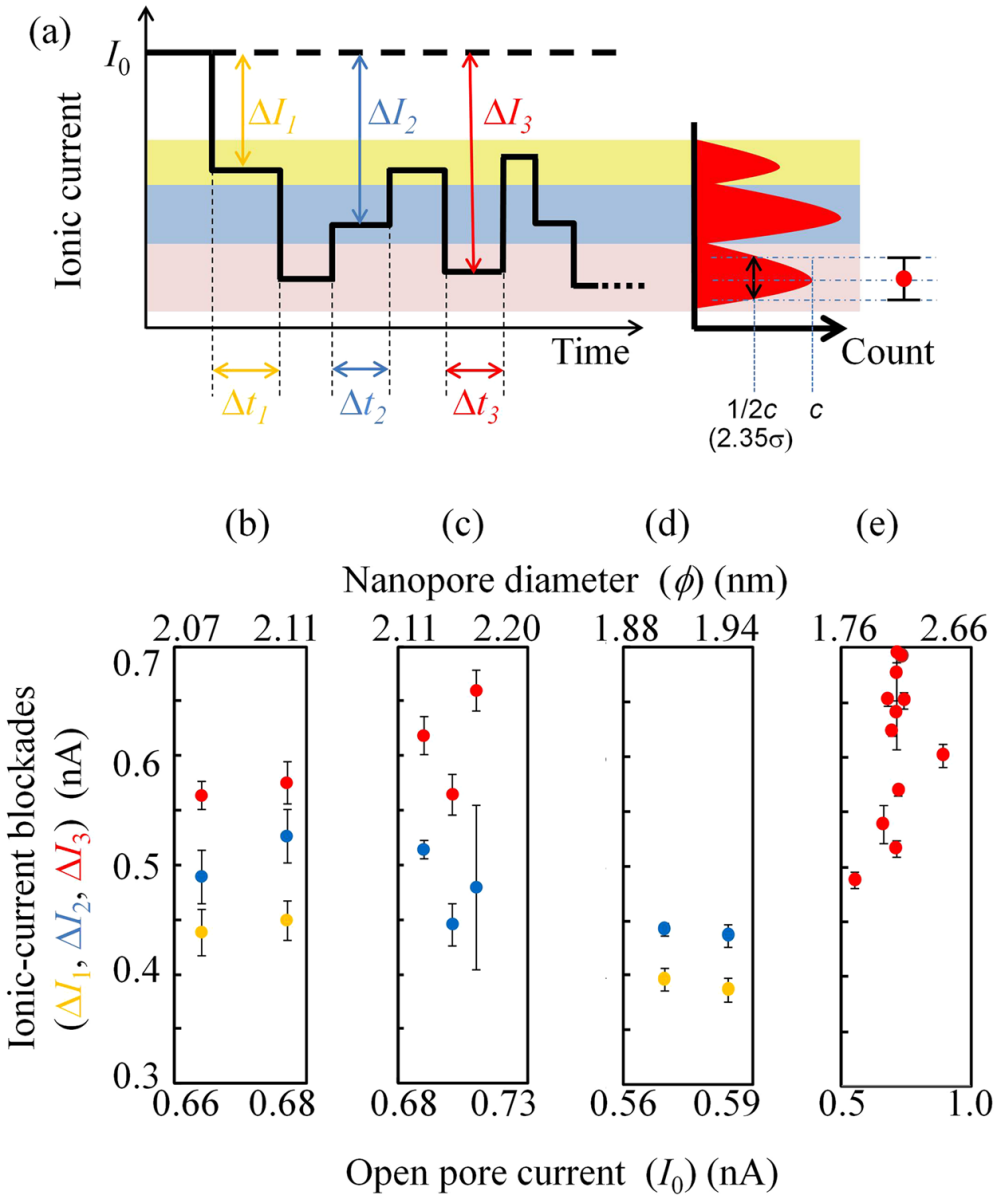
Relationship between the open pore current and Δ*I*. (**a**) Schematic of the time trace of the ionic current while DNA remained in the nanopore. Δ*I*_1_ < Δ*I*_2_ < Δ*I*_3_ are represented in orange, blue and red, respectively. (**b**) Data for when [(dT)_25_-(dC)_25_-(dA)_50_]_m_ remained in the nanopore. (**c**) Data for when [(dA)_50_-(dC)_50_]_m_ remained in the nanopore. (**d**) Data for when [(dT)_25_-(dC)_25_]_m_ remained in the nanopore. (**e**) Data for when poly(dA)_5.3k_ remained in the nanopore.

To investigate whether each homopolymer in an ssDNA can be discriminated by differences in the ionic current through the nanopore, we prepared different probes; a different block copolymer, ([(dT)_25_-(dC)_25_-(dA)_50_]_m_, [(dT)_25_-(dC)_25_]_m_ or [(dA)_50_-(dC)_50_]_m_), was immobilized for each probe. The prepared block copolymers contain long polymers with m of greater than 100 (see Supplementary Information SI-8). The minimum unit of homopolymer length is approximately 15.8 nm (25 bases), which is greater than *h*_eff_ = 3.75 nm. Details of the method used to prepare these block copolymers are described in the Materials and Methods section. Figure 5 presents representative examples of 10-sec time traces of the ionic currents when each ssDNA (poly(dA)_5.3k_ in Fig. 5(a), [(dT)_25_-(dC)_25_]_m_ in Fig. 5(b), and [(dT)_25_-(dC)_25_-(dA)_50_]_m_ in Fig. 5(c)) remained in the nanopore. The behaviour of each ionic current in each context differs from that in other contexts. For example, compared with the results shown in Fig. 5(a) in which a single level of ionic-current was observed, in the experiments shown in Fig. 5(b) and (c), more than one level of ionic-current was observed. When these data were obtained, the actuators were not moving the probe. In addition, the data in Figs 6 and 7 were also obtained while the probe was not actuated.

Figure 6 presents representative 1-sec time traces of ionic currents for (a) poly(dA)_5.3k_, (b) [(dT)_25_-(dC)_25_]_m_, (c) [(dA)_50_-(dC)_50_]_m_, and (d) [(dT)_25_-(dC)_25_-(dA)_50_]_m_, and the histograms of the detected currents. The diameters of each nanopore are as follows: (a) 2.07 nm, (b) 1.93 nm, (c) 2.13 nm, and (d) 2.08 nm. In the histograms, a single peak was confirmed for the measurement of poly(dA)_5.3k_, double peaks were confirmed for the measurements of [(dA)_50_-(dC)_50_]_m_ and [(dT)_25_-(dC)_25_]_m_, and triple peaks were confirmed for the measurements of [(dT)_25_-(dC)_25_-(dA)_50_]_m_. These findings indicate that the ionic current observed can have different current levels that correspond to the number of different homopolymers in an ssDNA. The consideration of the possible reasons why such multiple peaks were observed while the probe was not moved by the actuators was described in the Discussion section.

Figure 7 shows the relationships between the values of the open pore currents (*I*_0_) observed and the values of the ionic-current blockades (Δ*I*). Here, Δ*I* is defined as the current at the intensity peak in the current histograms. As described above, multiple Δ*I* values appear (Fig. 7(a)), and different Δ*I* levels are represented using the different-colour plots in Fig. 7(b–e). The width of error bar of each plot was determined as the FWHM (full width at half maximum; 2.35σ) of the Gaussian fitting curve to each Δ*I* histogram.

Figure 7(b) presents the three different levels of Δ*I* while [(dT)_25_-(dC)_25_-(dA)_50_]_m_ was in the nanopore. Figure 7(c) shows the two different levels of Δ*I* while [(dA)_50_-(dC)_50_]_m_ remained in the nanopore. Figure 7(d) depicts two different levels of Δ*I* observed while [(dT)_25_-(dC)_25_]_m_ remained in the nanopore. Figure 7(e) presents the single level of Δ*I* observed while poly(dA)_5.3k_ remained in the nanopore.

A comparison of these figures indicates that the values of the red data points in Fig. 7(b) and (c) are similar to those in Fig. 7(e). Therefore, the red data points in Fig. 7(b) and (c) are also assumed to represent Δ*I* caused by (dA)_n_. In addition, when (dT)_n_ was included in an ssDNA, Δ*I* values less than approximately 0.44 nA were observed, indicating that the orange data points in Fig. 7(b) and (d) represent Δ*I* caused by (dT)_n_. Finally, the remaining blue data points in the figure are assumed to represent Δ*I* caused by (dC)_n_. It would be good to perform experiments using probes on which poly(dC)_5.3k_ and poly(dT)_5.3k_ are immobilized for further verification of the correspondence between Δ*I* and the type of homopolymers. However, we could not prepare such DNA samples. We tried to prepare long ss-poly(dT) from ds-poly(dA)-poly(dT) using a similar method to that used to make ss-poly(dA) from ds-poly(dA)-poly(dT). The gel-electrophoresis result of the prepared ss-poly(dT) is presented in Supplementary Information SI-9 (Fig. SI-9(a)). The bandwidth of the created ss-poly(dT) was so broad that adequate amounts of long ss-poly(dT) could not be obtained. As for ss-poly(dC), we tried to prepare it from ds-poly(dG)-poly(dC). The gel-electrophoresis result of ds-poly(dG)-poly(dC) is presented in Supplementary Information SI-9 (Fig. SI-9(b)). Only short-length ds-poly(dG)_m_-poly(dC)_m_ (m < 1000) were created although several reaction temperatures were examined to elongate the ds-poly(dG)-poly(dC). In addition, long ss-poly(dC) and ss-poly(dT) cannot be created by using the rolling circle amplification (RCA) reaction which was used for the preparations of [(dT)_25_-(dC)_25_]_m_, [(dA)_50_-(dC)_50_]_m_, and [(dT)_25_-(dC)_25_-(dA)_50_]_m_ because ss-poly(dG) and ss-poly(dA) cannot be circularized.

The voltage dependency of the ionic current through the nanopore when [(dT)_25_-(dC)_25_-(dA)_50_]_m_ remained in the nanopore is presented in Supplementary Information SI-10. Δ*G* (=Δ*I*/*V*) derived from each homopolymer did not vary significantly depending on the applied voltage, which also indicates that each current-blockade level was derived from each homopolymers. The dwell times (Δ*t*) at different Δ*I* levels when [(dA)_50_-(dC)_50_]_m_, [(dT)_25_-(dC)_25_]_m_, and [(dT)_25_-(dC)_25_-(dA)_50_]_m_ remained in nanopores are presented in Supplementary Information SI-11. When [(dA)_50_-(dC)_50_]_m_ remained in the nanopore, the histogram of Δ*t* at the (dA)_50_ level was almost the same as that found at the (dC)_50_ level. However, from the histogram of Δ*t* when [(dT)_25_-(dC)_25_]_m_ remained in the nanopore, dwell times at the (dC)_25_ level were longer than those at the (dT)_25_ level. In addition, when [(dT)_25_-(dC)_25_-(dA)_50_]_m_ remained in the nanopore, the histogram of Δ*t* at the (dA)_50_ level was almost the same as that found at the (dT)_25_ level. Consequently, the correlation between the length of each block homopolymer and the dwell time was not confirmed in our experiment.

**Figure 8:**
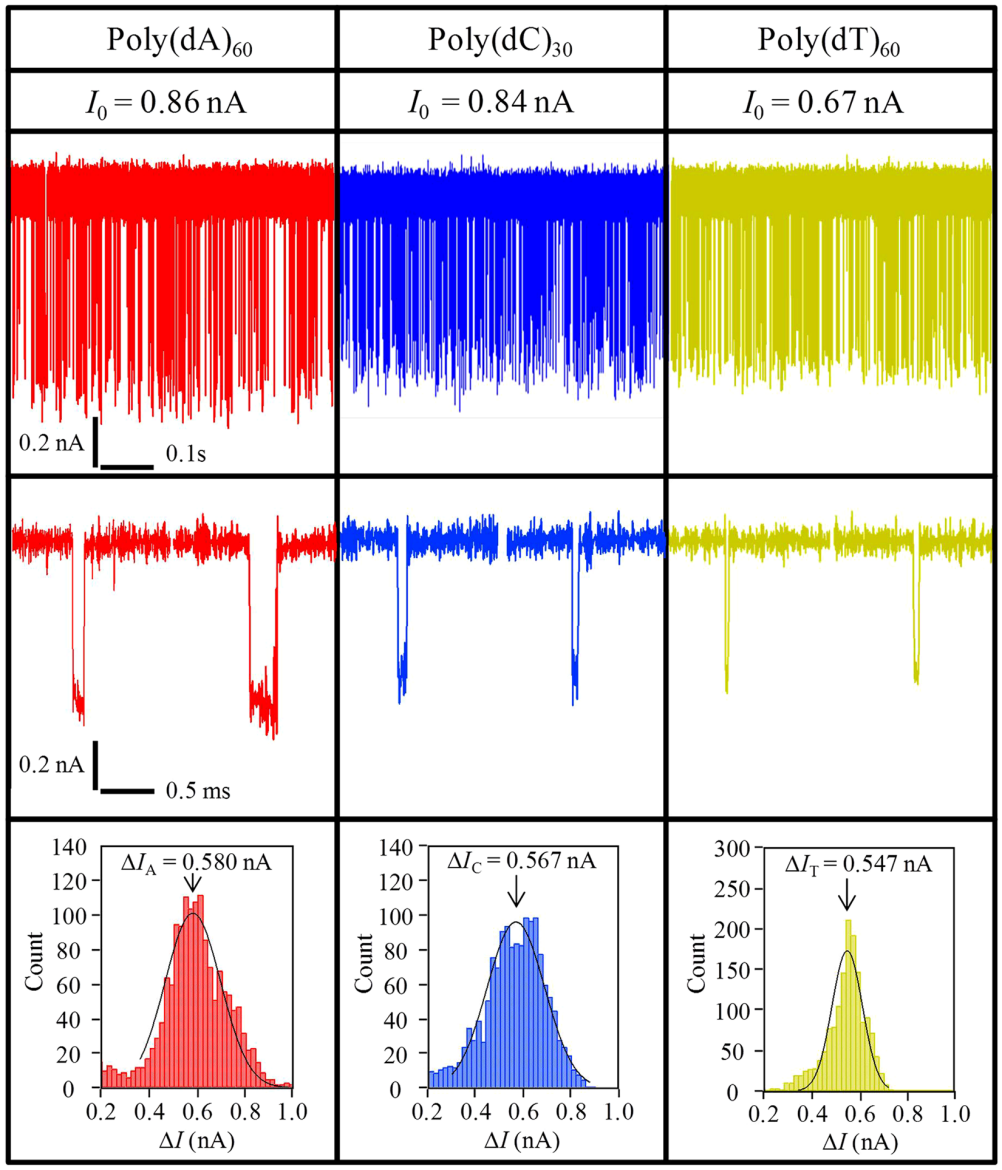
Ionic-current blockades when free poly(dA)_60_, poly(dC)_30_, and poly(dT)_60_ passed through a nanopore. Ionic-current blockades (Δ*I*) and their histograms when poly(dA)_60_, poly(dC)_30_, and poly(dT)_60_ passed through a nanopore. The aqueous solution in the *cis* chamber was 1 M KCl with 100 nM poly(dA)_60_, poly(dC)_30_ or poly(dT)_60_. The aqueous solution in the *trans* chamber was 1 M KCl. The applied voltage was 0.1 V. This measurement was performed with the same nanopore. The order of the measurement is (1) poly(dT)_60_, (2) poly(dC)_30_, (3) poly(dA)_60_. The nanopore was washed with pure water between each measurement. The open pore current (*I*_0_) gradually increased with nanopore washing and changing samples, which was difficult to suppress. The peak value of Δ*I* differed in each histogram.

The comparison of these conductance-blockade data with those obtained when free ssDNA passed through nanopores is shown in Table 1. In this table, all data, including those from two previous experiments^11, 35^, were acquired using Si_3_N_4_ nanopores and 1 M KCl aqueous solutions. Δ*G* values when free ssDNA passed through nanopores are listed in the top three rows. We acquired the data in the third row. Figure 8 shows the ionic current blockades and their histograms when free poly(dA)_60_, poly(dC)_30_ and poly(dT)_60_ passed through our nanopore. This experiment was performed by using the same nanopore chip shown in Fig. 1(c). A polyimide film was coated around the membrane to reduce the electrical noise. The order of the measurement is (1) poly(dT)_60_, (2) poly(dC)_30_, (3) poly(dA)_60_. The nanopore was washed with pure water between each measurement. Δ*G* values when ssDNA immobilized on the probe (i.e., tethered ssDNA) remained in nanopores are listed at or below the fourth row. All data acquired in this study are listed. The rank of Δ*G* when tethered ssDNA remained in nanopores was consistent with our result when free ssDNA passed through a nanopore. However, the rank of Δ*G* differed among the three results regarding free ssDNA translocations. The differences in Si_3_N_4_ membrane thickness, the process of nanopore fabrication, the formation conditions of the Si_3_N_4_ membrane, and the sampling frequency of the measurement tool may contribute to this discrepancy. However, we do not have evidence available at the moment to explain this discrepancy.

**Table 1:** Conductance blockades (Δ*G*) when each homopolymer or block copolymer passed through or remained in nanopores.

DNA motion control	Sample	Δ*G*(dA) (pS)	Δ*G*(dC) (pS)	Δ*G*(dT) (pS)	Voltage (mV)	Δ*G*_MAX_ *-*Δ*G*_MIN_ (pS)	Rank of Δ*G*	Reference
Free	(dA)_30_, (dC)_30_, (dT)_30_	5100	4200	4800	1000	900	A > T > C	Venta *et al*.^11^
Free	(dA)_40_, (dC)_40_, (dT)_40_	3510	3670	3800	200	290	T > C > A	Lee *et* *al*.^35^
Free	(dA)_60_, (dC)_30_, (dT)_60_	5800	5670	5470	100	330	A > C > T	Our result (Fig. 8)
Tethered	[(dT)_25_(dC)_25_]_m_	—	4420	3920	100	500	C > T	This work
Tethered	[(dT)_25_(dC)_25_]_m_	—	4370	3870	100	500	C > T	↑
Tethered	[(dA)_50_(dC)_50_]_m_	6540	4740	—	100	1800	A > C	↑
Tethered	[(dA)_50_(dC)_50_]_m_	6180	5130	—	100	1050	A > C	↑
Tethered	[(dA)_50_(dC)_50_]_m_	5630	4460	—	100	1170	A > C	↑
Tethered	[(dT)_25_(dC)_25_(dA)_50_]_m_	5780	5240	4500	100	1280	A > C > T	↑
Tethered	[(dT)_25_(dC)_25_(dA)_50_]_m_	5630	4900	4360	100	1270	A > C > T	↑
Tethered	[(dA)_50_(dC)_50_]_m_	7690	5400	—	200	2290	A > C	↑
Tethered	[(dA)_50_(dC)_50_]_m_	6100	4820	—	200	1280	A > C	↑
Tethered	[(dT)_25_(dC)_25_(dA)_50_]_m_	5910	5300	4980	200	930	A > C > T	↑
Tethered	[(dT)_25_(dC)_25_(dA)_50_]_m_	5840	5210	4930	300	910	A > C > T	↑

Δ*G* was defined as the peak value of Gaussian fits to each histogram. All data (including the data from two previous studies) were acquired using Si_3_N_4_ nanopores and in 1 M KCl aqueous solution. Δ*G* values obtained during the free (untethered) ssDNA translocations through nanopores are listed in the top three rows. Δ*G* values obtained when tethered ssDNAs remained in nanopores are listed at or below the fourth row.

Compared with the results when free ssDNA passed through nanopores, the differences between each Δ*G* derived from each homopolymer were greater when tethered ssDNAs remained in nanopores. This is thought to be due to the difference in the formation of ssDNA around the nanopore, which is explained in the Discussion section in detail.

Δ*G* when free poly(dA)_5.3k_ passed through a nanopore was 5860 pS (see Supplementary Information SI-12), which is consistent with the result in the case of poly(dA)_60_ translocations. The behaviour of ionic-current blockade when free [(dA)_50_-(dC)_50_]_m_ passed through a nanopore was also confirmed (see Supplementary Information SI-13). Although two current-blockade levels were infrequently observed in one translocation event, the split between the two levels was not clear in most events.

The ionic current blockades and the histogram of their dwell times at 300 mV while being pulled [(dT)_25_-(dC)_25_-(dA)_50_]_m_ by the actuator are presented in Supplementary Information SI-14. Compared with the histograms of the dwell times at 100 and 200 mV when the probe was not moved, the dwell times at the (dA)_50_ level were reduced. However, no significant decreases in the dwell times at the (dT)_25_ and (dC)_25_ levels were confirmed. In addition, the measured ionic-current while pulling the probe did not exhibit the ideal repeated-step signal.

## Discussion

We examined the possibility of discriminating each homopolymer in the ssDNA using solid-state nanopores and DNA-immobilized probes with actuators. To easily insert DNA into a nanopore without scanning for the location of the nanopore, a probe was prepared with a tip that was flat, square and larger than the area of the membrane. The surface of an oxidized Si substrate was used as the tip of the probe, and ssDNAs were immobilized on the surface at high density through use of peptide binding.

Ionic-current blockades were observed when the poly(dA)_5.3k_-immobilized probe approached the membrane, and the blocked current recovered to the original current value (i.e., the value of the open pore current) when the probe was moved far from the membrane (Fig. 3(a) and (b)). This indicated that immobilized DNA could be pulled in and out of the nanopore using our actuating system. Our nanopore chip has a thick SiO/Si_3_N_4_ layer (=350 nm) around the nanopore, indicating that ssDNA could be captured when the distance between the probe and the nanopore was greater than 350 nm. In order to investigate the capture radius of the nanopore, it is effective to set the sensor which measures the distance between the probe and the membrane while sensing the ionic current through the nanopore. This investigation will be addressed in future research.

**Figure 9:**
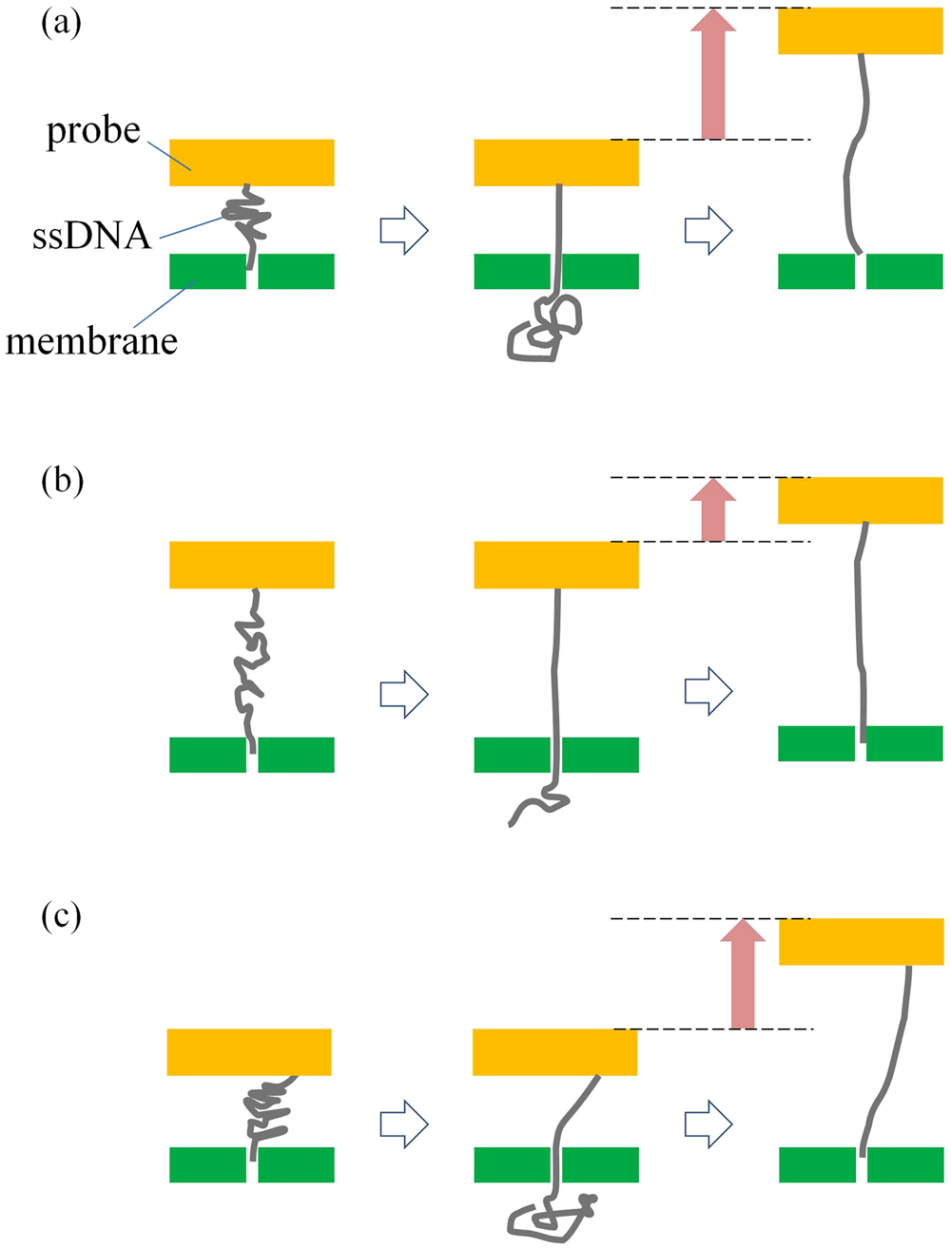
Different examples of how immobilized DNA might enter the nanopore. (**a**) An example of when a tightly folded DNA enters the nanopore and the DNA is subsequently pulled out. (**b**) An example of when a less-folded DNA enters the nanopore and the DNA is subsequently pulled out. (**c**) An example of the situation that occurs when the nanopore is not aligned to the point at which DNA is attached to the probe.

The time required to pull poly(dA)_5.3k_ out of the nanopore (*t*_out_) varied within each pull-out speed. One possible reason for this variability is the variation in the length of ss-poly(dA)_5.3k_. The variation in the number of bases of poly(dA)_5.3k_ was approximately 5.3-kb ± 0.4-kb. Another possible reason for this variability is the variation in the time between the observation of ionic-current blockade and when the probe movement was stopped, this time lag was thought to be up to one second. However, we believe that this is not the main reason for the variability, because the variation in *t*_out_ was greater than 10 sec. A third possible reason is the variation in the timing of when the DNA was pulled into the nanopore. Such variability can be caused by the difference in the formation of the DNA on the probe (Fig. 9(a,b)). If DNA is complexly folded (Fig. 9(a)), then the tip of the DNA can enter the nanopore when the probe is close to the membrane. In this context, a large part of DNA can pass through the nanopore once the tip of the DNA enters the nanopore. However, if the DNA is less folded (Fig. 9(b)), then the tip of the DNA can enter the nanopore even if the probe is not close to the membrane. In this case, only a small part of the DNA passes through the nanopore. Consequently, the variation in *t*_out_ is caused by the difference between these two different scenarios. A fourth possible reason is the variation in the alignment between the nanopore and the point where the DNA is attached to the probe (Fig. 9(c)). In this situation, the length of the DNA passing through the nanopore can vary even when the distance between the probe and the membrane is the same.

We measured ionic currents while several block copolymers remained in the nanopore, and found that the number of levels of current observed corresponded to the number of the different homopolymers present in the ssDNA. These signals could be obtained even when the probe speed was set to zero. In addition, the ideal repeated-step signal was not obtained while actuating the probe and pulling the ssDNA. Therefore, we assume that the following factors caused the fluctuation in the DNA position in the nanopore: (i) Brownian motion of the DNA; (ii) interaction forces between the DNA and the nanopore and between DNA and the surface of the membrane; (iii) electroosmotic flow (EOF) that is generated at the surface of the nanopore; and (iv) fluctuation of the distance between the probe and nanopore. The Brownian motion of DNA in a Si_3_N_4_ nanopore was theoretically estimated by Lu *et al*.^36^, who predicted that the position of each nucleotide in DNA can fluctuate approximately in the range of ±1 nm during DNA translocation through a Si_3_N_4_ nanopore at 0.1 V.

Regarding the interaction force between DNA and Si_3_N_4_, we measured the force using AFM, and the attraction force was observed (see Supplementary Information SI-15). Considering that the surface of Si_3_N_4_ film is negatively charged in our aqueous solution (pH = 7.5)^37^, the phosphate-backbone side of DNA is unlikely to attach to the Si_3_N_4_ surface because DNA has negative charges in its backbone side. Consequently, the attraction force was thought to be generated between the base side of ssDNA and the Si_3_N_4_ surface. When DNA was within the nanopore, other forces due to electric field and EOF also acted on the DNA. As a result, the DNA could stick and unstick from the surface of the nanopore, which could cause fluctuations of the DNA motion.

Regarding EOF, the direction of the flow is opposite to that of the force acting on the DNA via the electric field because the surface of Si_3_N_4_ is negatively charged. Several previous studies reported that EOF reduces the translocation time and entry rate of DNA into a nanopore^38–40^. The effect of EOF is large at the surface of the nanopore. In our case, the tethered DNA might move in the inverse direction when the DNA is close to the surface of the nanopore.

The fluctuation of the distance between the probe and nanopore should also be considered. As described above, the fluctuation in the relative distance between the probe and nanopore is assumed to be up to 3–4 nm.

Comparison of the ionic-current blockade levels (Δ*I*) obtained from poly(dA)_5.3k_, [(dT)_25_-(dC)_25_]_m_, [(dA)_50_-(dC)_50_]_m_, and [(dT)_25_-(dC)_25_-(dA)_50_]_m_ suggests that the highest Δ*I* corresponds to (dA)_n_, the second highest Δ*I* corresponds to (dC)_n_, and the lowest Δ*I* corresponds to (dT)_n_. The value of Δ*I* from each homopolymer has a variation. Several possible causes contribute to this variation. For example, variations in the effective thickness or geometry of the nanopore can contribute to the variation in Δ*I*. Moreover, the angle at which the DNA enters the nanopore might also contribute to the variation.

We compared these conductance-blockade levels with those observed when free ssDNA passed through nanopores. The rank of Δ*G* when tethered ssDNA remained in a nanopore was consistent with our result obtained when free ssDNA passed through a nanopore. When tethered ssDNA remained in a nanopore, the differences between each Δ*G* derived from each homopolymer were greater than those when free ssDNA passed through a nanopore. To consider this, we focused on the difference in the formation of ssDNA around the nanopore when free ssDNA passes through or tethered ssDNA remains in a nanopore. When free ssDNA passes into a nanopore, ssDNA is so flexible that it can be coiled in the access resistance region, which causes the significant contribution to Δ*G* (the access resistance is expressed in 1/*ϕ* terms in Equation (1)). In addition, the formation of the coil changes over time. These factors tend to hide the true value of Δ*G* derived from each homopolymer. In contrast, when tethered ssDNA remains in a nanopore, the ssDNA is thought to be rather stretched around the access region of the nanopore because the two forces acting in the opposite direction (i.e., the force by electric field in the nanopore and the binding force between ssDNA and the probe) acted on the ssDNA. As a result, the differences between each Δ*G* derived from each homopolymer became clearer.

It is noted that Δ*G* observed when free poly(dA) passed through an α-hemolysin channel was also greater than that observed when free poly(dC) passed through it^41^. This trend is consistent with our results. Regarding the dwell time (Δ*t*) of each homopolymer in the α-hemolysin channel, Δ*t* of free poly(dA) was greater than that of free poly(dC). It cannot be simply compared with our result regarding Δ*t* because our experiments used tethered DNAs. In our result, the histogram of Δ*t* at the (dA)_50_ level was almost the same as that found at the (dC)_50_ level when [(dA)_50_-(dC)_50_]_m_ remained in the nanopore (Figure SI-11(f) in Supplementary Information SI-11).

We believe that the findings in this study further support the possibility of DNA sequencing with solid-state nanopores.

## Methods

### Fabrication of solid-state nanopores

The methods for fabricating the membranes and nanopores followed the report by Yanagi *et al*.^31^ Nanopores were fabricated in a 10-nm-thick silicon nitride (Si_3_N_4_) membrane using the multilevel pulse voltage injection (MPVI) technique. Prior to nanopore fabrication, both sides of the Si_3_N_4_ thin membrane were cleaned with Ar/O_2_ plasma (10 W, 20 sccm, 20 Pa, 45 sec) (SAMCO Inc., Kyoto, Japan) and the membrane was mounted on a custom-built acrylic flow cell. Next, the two chambers formed in the flow cell were filled with a 1 M KCl and 1 mM Tris-10 mM EDTA (pH 7.5) electrolyte, and Ag/AgCl electrodes were placed in both sides of the chambers.

In the MPVI technique, the voltage pulses to form a nanopore were set at the same voltages as those reported in ref. 31. The voltage pulses were generated with a 41501B SMU AND Pulse Generator Expander (Agilent Technologies, Inc., Santa Clara, CA). The current after each voltage pulse was measured using a 4156B Precision Semiconductor Analyzer (Agilent Technologies, Inc., Santa Clara, CA).

### Preparation of the block copolymers

For preparation of the DNA-immobilized probes, the end of a single-stranded DNA was modified with NH_2_ and the NH_2_-terminal-modified DNA was immobilized on an oxidized Si substrate coated with APTES and glutaraldehyde (5%, Nishin EM, Inc., Tokyo, Japan). The immobilized DNAs were NH_2_-poly(dA)_5.3k_ or block copolymers comprising two or three homopolymer subunits, namely: NH_2_-[(dA)_50_-(dC)_50_]_m_, NH_2_-[(dT)_25_-(dC)_25_]_m_, or NH_2_-[(dT)_25_-(dC)_25_-(dA)_50_]_m_. The NH_2_-poly(dA)_5.3k_ was prepared by following the preparation method reported in ref. 24 using an NH_2_-dA_20_ primer. The block copolymers were prepared using two reaction steps. In the first step, a DNA template was circularized using CircLigase^TM^ ssDNA Ligase (Epicentre., Madison, WI), which catalysed the intermolecular ligation (i.e., circularization) of the ssDNA that had a 5′-monophosphate and a 3′-hydroxyl group. Each DNA template was purchased from Sigma-Aldrich Japan, Inc. The reaction mixture contained 1x CircLigase ssDNA Ligase Reaction Buffer, 10 pmol 5′-monophosphate DNA template, 2.5 mM MnCl_2_, 1 M betaine, and 0.25 U/μL CircLigase. A DNA template was circularized by keeping the reaction mixture at 60 °C overnight followed by denaturation of the enzyme at 80 °C for 20 min.

In the second step, a target block copolymer was synthesized by the rolling circle amplification (RCA) reaction. The reaction mixture of 20 μL contained 1x Phi29 Reaction Buffer, 200 μg/mL BSA, 0.1 U/μL Phi29 DNA polymerase (New England Biolabs Inc., Ipswich, MA), 1 μM RCA primer, 400 μM dNTP, and the circularized 1 μL DNA template. The RCA reaction was performed by keeping the reaction mixture at 30 °C overnight followed by denaturation of the enzyme at 80 °C for 20 min. The products of the RCA reaction (i.e., the target block copolymers) were monitored by 0.8% alkaline-agarose-gel electrophoresis using 2 μL of the reaction mixture. The combination of the primer, dNTP and DNA template was determined in accordance with each target block copolymer as shown in Table 2.

**Table 2:** The combination of DNA template, primer and dNTP used to prepare the block copolymer.

ID	Target block copolymer	Circularization	RCA reaction	
		DNA template	Primer	dNTP
1	NH_2_-[(dA)_50_-(dC)_50_]_m_	(dG)_50_-(dT)_50_	NH_2_-(dA)_20_	dATP, dCTP
2	NH_2_- [(dT)_25_-(dC)_25_]_m_	(dG)_25_-(dA)_25_	NH_2_-(dT)_20_	dTTP, dCTP
3	NH_2_- [(dT)_25_-(dC)_25_-(dA)_50_]_m_	(dG)_25_-(dA)_25_-(dT)_50_	NH_2_-(dA)_20_	dATP, dCTP, dTTP

### Immobilization of NH_2_-modified DNA

For preparation of the DNA-immobilized probe, an APTES monolayer was formed on the oxidized surface of the Si wafer at a thickness of 725-μm (300 mTorr, 55 °C, MVD-100, AMST Inc., San Jose, CA). The wafer was diced into chips with areas of 1.4 mm × 1.4 mm. The chips were dipped in a 5% solution of glutaraldehyde (Nishin EM, Tokyo, Japan) and sodium cyanoborohydride (4 mM, Tokyo Kasei, Japan) in phosphate-buffered solution (PBS, GE Health Care, Chicago, IN) for 40 min and were then rinsed in PBS. Then, the chip was immersed in the solution composed of the reaction mixture containing NH_2_-modified DNA (this is explained in the Methods section which describes the “Preparation of block copolymers”) and sodium cyanoborohydride (4 mM in PBS, Tokyo Kasei, Japan) for 40 min followed by rinsing with diluted water. At this point, the NH_2_-modified DNA was immobilized on the surface of the probe by peptide binding between glutaraldehyde and NH_2_. Sodium cyanoborohydride (4 mM in PBS, Tokyo Kasei, Japan) was used to reduce the imine bond and stabilize the reaction. Prior to the nanopore measurements with DNA, the probe was exposed to pure water for one hour at 60 °C to reduce the unreacted and folded DNA and the probe was stored in pure water at 4 °C.

### Fluorescence observation of DNA, APTES and glutaraldehyde

Observations were performed using a laser scanning confocal microscope (LSM 780, Carl Zeiss, Oberkochen, Germany). Fluorescent dyes were selected to correspond to the targets for observation on the surface of the probe. Alexa Fluor® 488 5-SDP Ester (Thermo Fisher Scientific Inc., Waltham, MA) was used to observe APTES, Alexa Fluor® 488 Cadaverine (Thermo Fisher Scientific Inc., Waltham, MA) was used to observe glutaraldehyde, and SYBR® Gold Nucleic Acid Gel Stain was used to observe DNA. Labelling APTES or glutaraldehyde with Alexa Fluor® 488 5-SDP ester or Alexa Fluor® 488 Cadaverine was performed as follows: A solution that contained pure water, NaHCO_3_ (100 mM) and Alexa Fluor® 488 5-SDP ester or Alexa Fluor® 488 Cadaverine (8:1:1) was dropped onto the surface of the probe and incubated at room temperature for one hour. To prevent the droplet on the chip from drying, the chip was covered with a CoverWell^TM^ Perfusion Chamber (PC8R-2.0, Grace Bio-Labs, Inc., Bend, OR). The chip was subsequently rinsed with pure water and PBS (pH 7.4). Labelling DNA with SYBR® Gold was performed as follows. The probe was immersed in a 0.1% solution of SYBR® Gold in TAE buffer and shaken at 45 rpm for 40 min. The chip was subsequently rinsed with pure water and PBS (pH 7.4).

### Setup for nanopore measurement with a DNA-immobilized probe

The DNA-immobilized probe was mechanically clamped to the piezo actuator (NPZ25-206, nPoint Inc., Middleton, WI). The stepper motor (AR15SAKD, ORIENTAL MOTOR Co., Ltd., Tokyo, Japan) was set on the piezo actuator. The probe was set into the channel in the *cis* chamber, which had a 2.5 mm diameter. For the measurements of the ionic currents through the nanopore except the result in Fig. 8, a patch clamp amplifier (Axopatch 200B, Axon instruments, Union City, CA) was used. The output signals were filtered with a four-pole Bessel filter with a cut-off frequency of 2-10 kHz and then digitized with a DAQ AD converter (NI USB-6281 18-bit DAQ, National instruments, Austin, TX) at 50 kHz. For the measurement shown in Fig. 8, another patch clamp amplifier (VC100, Chimera Instruments, New York, NY, USA) was used. The output signals were filtered with a four-pole Bessel filter with a cut-off frequency of 100 kHz, and digitized at 4166.7 kHz. All measurements were performed at room temperature (22–25 °C).

## Electronic supplementary material

Supplementary Information
