# Supplementary material for: Discrimination of three types of homopolymers in single-stranded DNA with solid-state nanopores through external control of the DNA motion
Source: Sci Rep. 2017 Aug 22;7:9073. doi: 10.1038/s41598-017-08290-6 (PMC5567245; doi:10.1038/s41598-017-08290-6)
Supplement: Supplementary file 1 — Supplementary Information [file 41598_2017_8290_MOESM1_ESM.doc]

Supplementary Information for

Discrimination of three types of homopolymers in single-stranded DNA with solid-state nanopores through external control of the DNA motion

Rena Akahori *, Itaru Yanagi, Yusuke Goto, Kunio Harada, Takahide Yokoi, and Ken-ichi Takeda

Hitachi Ltd., Research and Development Group, Center for Technology Innovation – Healthcare, 1-280, Higashi-koigakubo, Kokubunji, Tokyo, 185-8601

The Supplementary Information includes the following:

SI-1. Image of the measurement system

SI-2. Displacement of the probe monitored by a laser displacement meter

SI-3. Frequency characteristics of the vibration of the probe

SI-4. Positional fluctuations of the probe and the membrane chip

SI-5. Fluorescence images of each step of the DNA immobilization process

SI-6. Ionic current though a nanopore when the probe without DNA approached the nanopore membrane

SI-7. Estimation of variation in effective thickness of the nanopore and its diameter

SI-8. Gel electrophoresis images of prepared single-stranded DNAs

SI-9. Gel electrophoresis images of ss-poly(dT) and ds-poly(dG)-poly(dC)

SI-10. Voltage dependency of ionic-current blockade when [(dT)_25_-(dC)_25_-(dA)_50_]_m_ remained in nanopores

SI-11. Analysis of dwell time at each Δ*I* level when [(dT)_25_-(dC)_25_]_m_, [(dA)_50_-(dC)_50_]_m_ and [(dT)_25_-(dC)_25_-(dA)_50_]_m_ remained in nanopores

SI-12. Ionic-current blockades when free poly(dA)_5.3k_ passed through a nanopore

SI-13. Ionic-current blockade during free [(dA)_50_-(dC)_50_]_m_ translocation through a nanopore

SI-14. Analysis of dwell times at each Δ*I* level with and without pulling the probe when [(dT)_25_-(dC)_25_-(dA)_50_]_m_ remained in nanopores

SI-15. Force measurement between ssDNA and the surface of Si_3_N_4_

SI-1. Picture of the measurement system

The image below depicts the setup for measurement of ionic current through the nanopore when the DNA-immobilized probe approaches the nanopore.


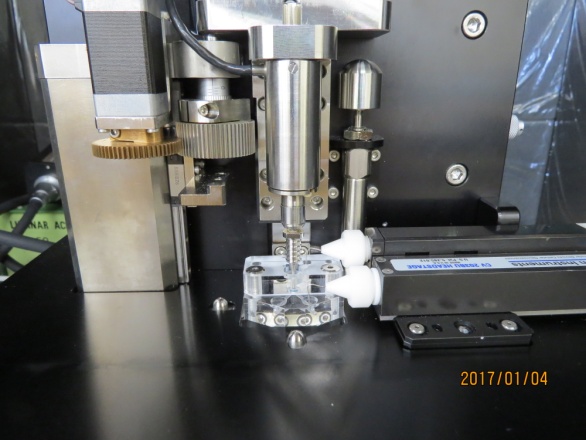


probe

stepper motor

piezo actuator

flow cell

ammeter


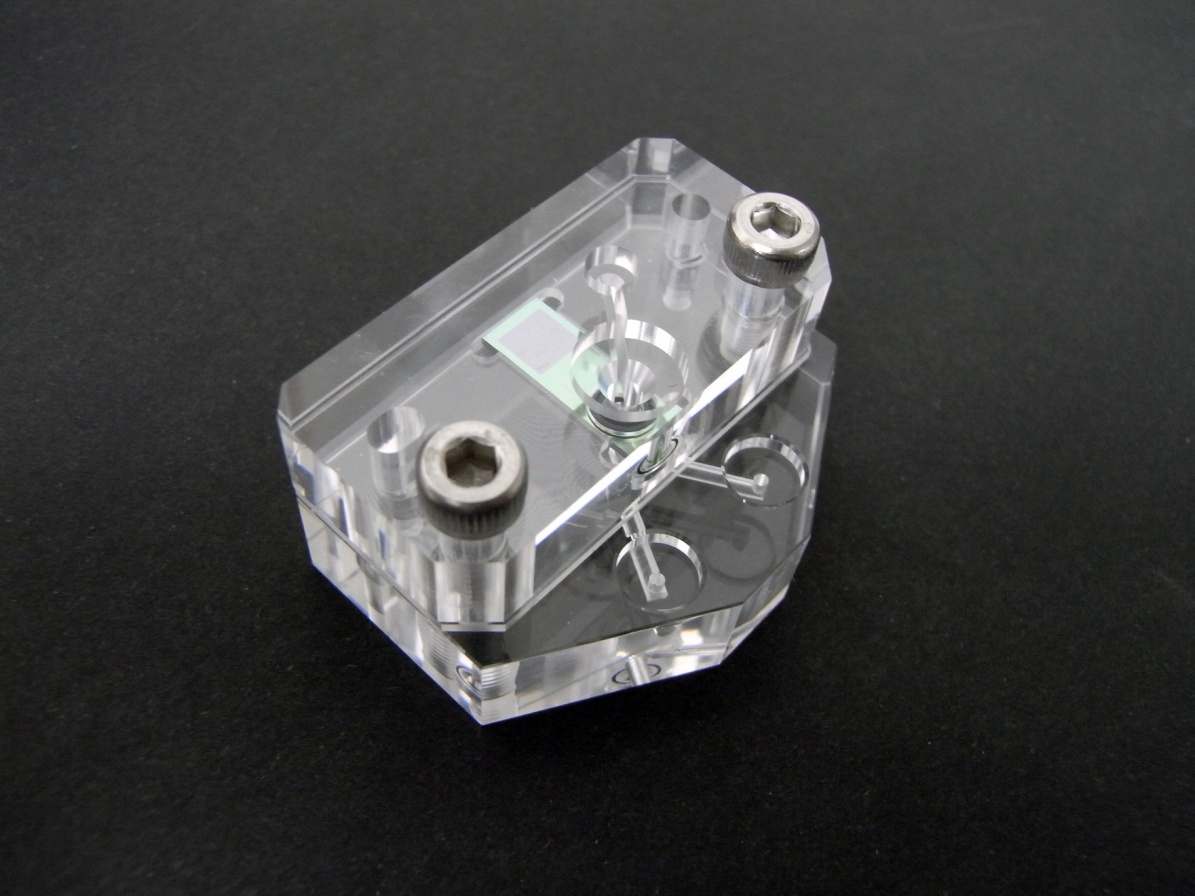

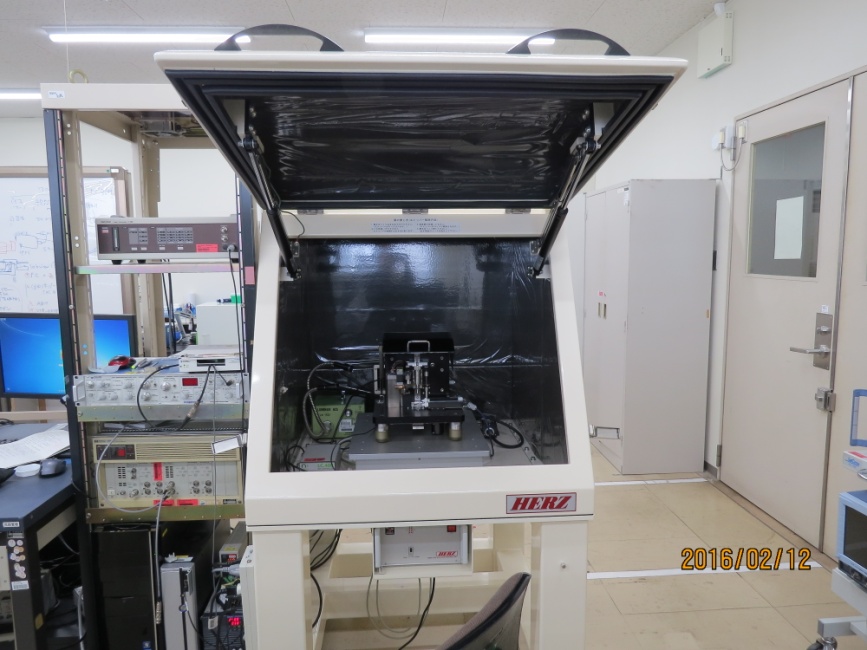


chip with nanopore

probe channel

acoustic enclosure

active vibration isolation system

Figure SI-1

SI-2. Displacement of the probe monitored by the laser displacement meter

Using a laser displacement meter (LV1800, ONO SOKKI CO., LTD., Kanagawa, Japan), we measured the displacement of the probe that was controlled by a piezo actuator. Figure SI-2(a) shows the time trace of the displacement of the probe when the velocities of the piezo actuator were set at ±340 nm/s. The measured velocities (i.e., the slopes of lines in the figure) were -340.50 nm/s and 340.11 nm/s. These values are in good agreement with the setting values of the piezo actuator. In addition, the hysteresis between the forward and backward movements was effectively suppressed by closed loop control of the piezo actuator.

Figure SI-2 (b) shows the time trace of the displacement of the probe when the velocity of the piezo actuator was set to zero. The probe crept with a velocity of approximately 0.486 nm/s.

Figure SI-2

SI-3. Frequency characteristics of the vibration of the probe

We measured the frequency characteristics of the vibration of the probe using the laser displacement meter. Figure SI-3(a) shows the frequency characteristics of the vibration intensities when the acoustic enclosure was open (represented by the red line) and closed (represented by the black line). The vibration of 500–600 Hz was effectively suppressed by the acoustic enclosure. Figure SI-3(b) presents the characteristics when the active vibration isolation system was not used (represented by the red line) and used (represented by the black line). The vibration of 10–100 Hz was effectively suppressed by the active vibration isolation system.


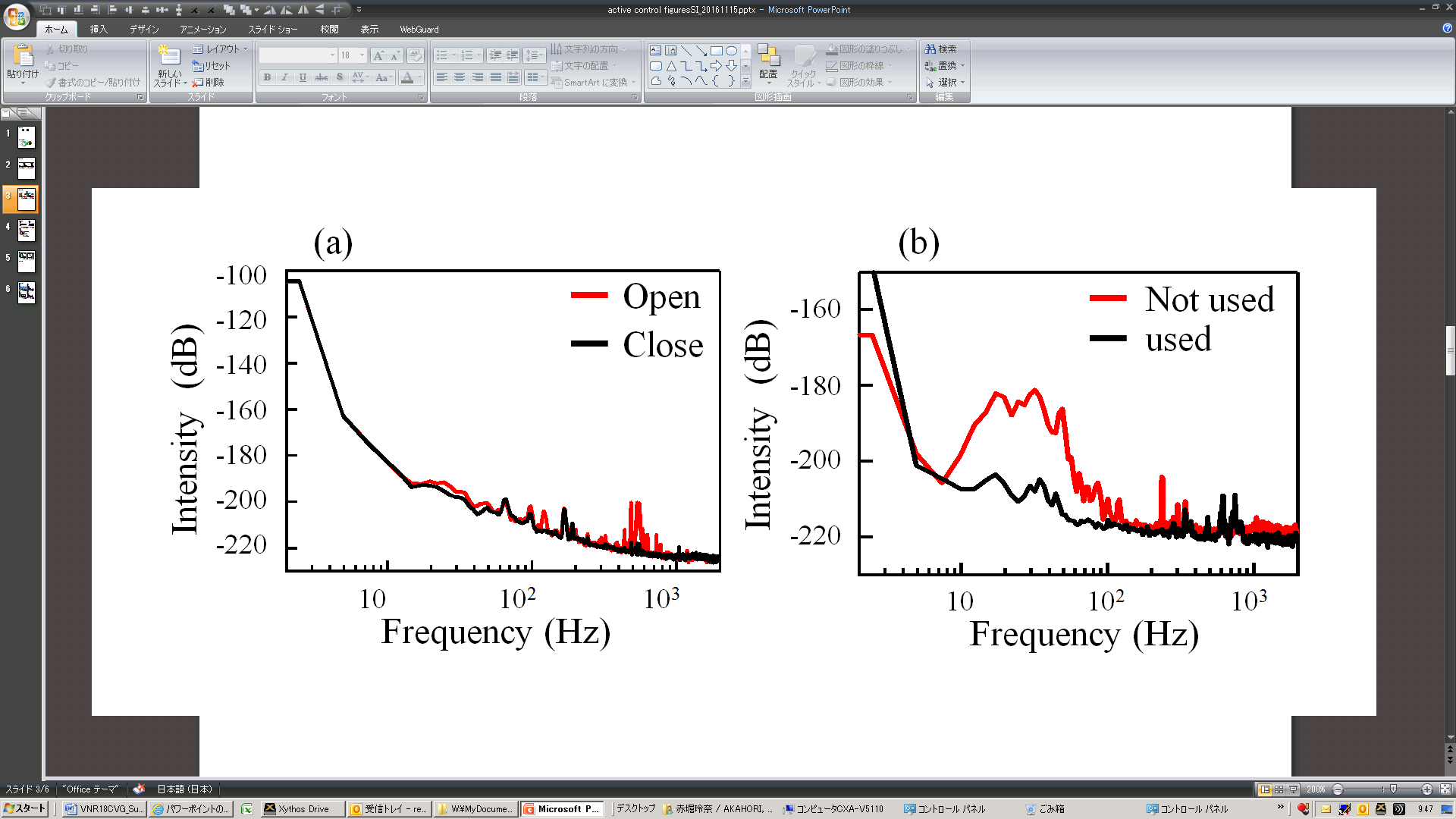


Figure SI-3

SI-4. Positional fluctuations of the probe and the membrane chip

We measured the positional fluctuations of the probe (Figure SI-4(a)) and the membrane chip (Figure SI-4(b)) when the speed of the actuator was set to zero. The measurements were performed using the laser displacement meter. The data reveal the possibility that both the probe and membrane fluctuate with a range of approximately 1-2 nm. Figure SI-4(c) shows a comparison of the frequency characteristics of the positional fluctuations. The frequency characteristic of the positional fluctuation of the probe (black line) is almost the same as that of the membrane chip (red line).

Figure SI-4

SI-5. Fluorescence images of each step of the DNA immobilization process

After APTES was formed on the surface of the probe, the fluorescence image of APTES was obtained using Alexa Fluor® 488 5-SDP ester (Figure SI-5(a)). Figure SI-5(b) shows the fluorescence image when APTES was not formed. These figures confirm that APTES could form on the probe. Figure SI-5(c) shows the fluorescence image of glutaraldehyde on APTES obtained using Alexa Fluor® 488 Cadaverine. Figure SI-5(d) shows the fluorescence image before glutaraldehyde was formed on the probe. These figures confirmed that a glutaraldehyde layer could form on APTES.

Figure SI-5

SI-6. Ionic current though a nanopore when the probe without DNA approached the nanopore membrane

Figure SI-6(a) shows the time trace of the ionic current through the nanopore when a probe on which DNA was not immobilized approached the membrane. The *cis* and *trans* chambers were filled with 1 M KCl. The applied voltage was 0.1 V. Figure SI-6(b) depicts the control voltage for the piezo actuation during the ionic-current measurement. In this experiment, the probe was moved by the stepper motor only. The motion by the piezo actuator was set to zero. Therefore, the control voltage should be constant unless any force acts on the piezo-electric element. Consequently, the change in the control voltage in Figure SI-6(b) indicated that the probe touched the membrane chip. The control voltage was recovered to the original value when the probe was moved away from the membrane. These results confirm that no ionic-current blockades occurred when the probe without DNA approached the membrane.

Figure SI-6

**SI-7. Estimation of variation in effective thickness of the nanopore and its diameter**

The relationship between the diameters of the nanopores (*φ*_m_) created using MPVI and the currents through the nanopores (*I*_0_) are presented in Figure SI-7(a) [1]. The *cis* and *trans* chambers were filled with 1 M KCl aqueous solutions. *I*_0_ was measured at 0.1 V. *φ*_m_ was approximated by an ellipsoidal approximation as *φ*_m_ = (*φ*_l_ × *φ*_s_)^1/2^, where *φ*_l_ and *φ*_s_ are the major and minor axes of nanopores measured from TEM images, respectively (Figure SI-7(b)). The calculation lines were drawn in accordance with the equation (1) in the main text. In our observations (nine plots in the graph), the calculation with *h*_eff_ = 3.75 nm is the central fitting line and the expected error is ±0.75 nm (shown as upper and lower lines).

The variation in nanopore diameter can also be estimated from the graph. Among the nanopores created for the experiments in the main text, *I*_0_=0.57 nA was the minimum open pore current, which corresponds to *φ*_m_=1.90±0.16 nm. *I*_0_=0.89 nA was the maximum open pore current in the main text, which corresponds to *φ*_m_=2.48±0.21 nm.

Figure SI-7

SI-8. Gel electrophoresis images of prepared single-stranded DNAs

The lengths of prepared single-stranded DNAs were analysed by alkaline-agarose-gel electrophoresis. Figure SI-8(a) presents the images of a size marker, ds-poly(dA)-poly(dT) (lane 1) and ss-poly(dA) (lane 2). The length of ss-poly(dA) was estimated as 5.3±0.4 kb [2]. Figure SI-8(b) presents the images of a size marker and [(dA)_50_-(dC)_50_]_m_ (lane 1). Figure SI-8(c) shows the images of a size marker and [(dT)_25_-(dC)_25_]_m_ (lane 1 to 4). Figure SI-8(d) presents the images of a size marker and [(dT)_25_-(dC)_25_-(dA)_50_]_m_ (lane 1 to 4). These figures confirm that prepared [(dA)_50_-(dC)_50_]_m_, [(dT)_25_-(dC)_25_]_m_ and [(dT)_25_-(dC)_25_-(dA)_50_]_m_ contain the long DNA samples (> 10 kilobases in length).

Figure SI-8

**SI-9.** **Gel electrophoresis images of ss-poly(dT) and ds-poly(dG)-poly(dC)**

We tried to make ss-poly(dT) from ds-poly(dA)-poly(dT) using a similar method to that used to make ss-poly(dA) from ds-poly(dA)-poly(dT) [2]. The gel electrophoresis images of a size marker and the prepared samples (i.e., ds-poly(dA)-poly(dT) (lane 1) and ss-poly(dT) (lane 2)) are presented in Figure SI-9(a). Unlike the case of ss-poly(dA), the constant length of ss-poly(dT) could not be prepared, and an adequate amount of long ss-poly(dT) was not obtained. Figure SI-9(b) shows the gel electrophoresis images of ds-poly(dG)-poly(dC) in lanes from 1 to 5. Unlike the case of ds-poly(dA)-poly(dT), only short-length ds-poly(dG)-poly(dC) was generated, although various reaction temperatures for elongation were examined (Table SI-9).

Figure SI-9

Table SI-9

| Lane | Reaction temperature (°C) | Sample |
| --- | --- | --- |
| 1 | 10 | ds-poly(dG)-poly(dC) |
| 2 | 20 |
| 3 | 30 |
| 4 | 40 |
| 5 | 50 |
|  |  |  |

SI-10. Voltage dependency of ionic-current blockade when [(dT)_25_-(dC)_25_-(dA)_50_]_m_ remained in nanopores

Figure SI-10(a)-(c) shows the representative 1-sec time traces of ionic currents when the block copolymer [(dT)_25_-(dC)_25_-(dA)_50_]_m_ remained in nanopores as well as histograms of the ionic currents. The applied voltages were (a) 0.1 V, (b) 0.2 V and (c) 0.3 V. Data were acquired using different nanopore membranes. The open pore currents and their estimated pore diameters are (a) *I*_0_=0.66 nA, *φ*=2.08 nm, (b) *I*_0_=1.31 nA, *φ*=2.07 nm, and (c) *I*_0_=2.38 nA, *φ*=2.31 nm. Table SI-10 presents Δ*I* and Δ*G* at different levels when the block copolymer remained in the nanopore at each voltage. Δ*I* and Δ*G* are defined as the peak value of Gaussian fits to each histogram.

Figure SI-10

Table SI-10

|  |  |  |  |  |  |  |
| --- | --- | --- | --- | --- | --- | --- |
| Applied Voltage (mV) | Δ*I*_A_  (nA) | Δ*I*_C_  (nA) | Δ*I*_T_  (nA) | Δ*G*_A_  (pS) | Δ*G*_C_  (pS) | Δ*G*_T_  (pS) |
| 100 | 0.56 | 0.49 | 0.44 | 5630 | 4900 | 4360 |
| 200 | 1.18 | 1.06 | 1.00 | 5910 | 5300 | 4980 |
| 300 | 1.75 | 1.56 | 1.48 | 5830 | 5210 | 4930 |
|  |  |  |  |  |  |  |

SI-11. Analysis of dwell time at each Δ*I* level when [(dT)_25_-(dC)_25_]_m_, [(dA)_50_-(dC)_50_]_m_ and [(dT)_25_-(dC)_25_-(dA)_50_]_m_ remained in nanopores

Figure SI-11(a) shows a schematic image of the ionic current when [(dT)_25_-(dC)_25_-(dA)_50_]_m_ remained in a nanopore. The histograms of ionic-current blockades can be fitted by three Gaussian curves. In the analysis in SI-11, the boundary current, which distinguishes one Δ*I* level from another, was defined at the cross point of two Gaussian curves. Figure SI-11(b)-(d) presents the scatter plots of Δ*I* and its dwell time (Δ*t*) when [(dT)_25_-(dC)_25_]_m_, [(dA)_50_-(dC)_50_]_m_, and [(dT)_25_-(dC)_25_-(dA)_50_]_m_ remained in nanopores. The histogram of Δ*t* corresponding to each scatter plot is presented in Figure SI-11(e)-(g). The diameters of the nanopores used in (b), (c) and (d) were 1.93 nm, 2.13 nm and 2.08 nm, respectively.

Figure SI-11

SI-12. Ionic-current blockades when free poly(dA)_5.3k_ passed through a nanopore

Figure SI-12(a) shows the time trace of the ionic current during free poly(dA)_5.3k_ translocation through a nanopore. The aqueous solution in *cis/trans* chamber was 1 M KCl with 1 nM poly(dA)_5.3k_/1 M KCl. The applied voltage was 0.1 V. Figure SI-12(b) presents magnified views of the ionic-current blockades. The histogram of Δ*I* is shown in Figure SI-12(c). The peak value was calculated from Gaussian fits to the histogram.

Figure SI-12

SI-13. Ionic-current blockade during free [(dA)_50_-(dC)_50_]_m_ translocation through a nanopore

Figure SI-13 presents the time trace of the ionic current when free [(dA)_50_-(dC)_50_]_m_ passed through a nanopore. The aqueous solution in the *cis* chamber was 1 M KCl with 0.8 nM [(dA)_50_-(dC)_50_]_m_. The aqueous solution in the *trans* chamber was 1 M KCl. The applied voltage was 1 V. This measurement was performed with a nanopore coated with amine-functionalized silica beads for deceleration of passing speed through the nanopore [3, 4]. Two current-blockade levels in one translocation event were infrequently observed (Figure SI-13(a)). However, in most events, the split between two levels was not clear (Figure SI-13(b)).

Figure SI-13

SI-14. Analysis of dwell times at each Δ*I* level with and without pulling the probe when [(dT)_25_-(dC)_25_-(dA)_50_]_m_ remained in nanopores

Figure SI-14(a)-(c) shows the scatter plots of Δ*I* and Δ*t* at each level when [(dT)_25_-(dC)_25_-(dA)_50_]_m_ remained in nanopores*.* The boundary current, which distinguishes one Δ*I* level from another, was defined in the same manner as that described in SI-11. The applied voltages were set at (a) 100 mV, (b) 200 mV, and (c) 300 mV. The velocities of the probe (*v*_p_) were set at (a) 0 nm/s, (b) 0 nm/s, and (c) 34 nm/s (direction of pull-out). Figure SI-14(d)-(f) presents the histogram of Δ*t* corresponding to each scatter plot.

Figure SI-14(g) presents the schematic of the ideal ionic-current signal while pulling [(dT)_25_-(dC)_25_-(dA)_50_]_m_ out from the nanopore at the velocity of 34 nm/s. Figure SI-14(h) presents the measured ionic-current signal at 300 mV while pulling [(dT)_25_-(dC)_25_-(dA)_50_]_m_ with *v*_p_ of 34 nm/s. The ideal repeated-step signal was not obtained.

Figure SI-14

SI-15. Force measurement between ssDNA and the Si_3_N_4_ surface

Figure SI-15 presents force-distance profiles between the probe and the Si_3_N_4_ surface on the Si substrate, which were measured using an atomic force microscope (MFP-3D, Asylum Research). Figure SI-15(a) presents a schematic of the measurement. The measurement was performed in 1 M KCl aqueous solution. The cantilever (AIO-TL, JEOL) with 0.2 N/m spring constant and 15 kHz resonant frequency was used. An amine-functionalized 20-μm bead was attached to the tip of the cantilever. The approaching and retreating speed was set at 1.98 μm/s. The force curves were measured repeatedly. Figure SI-15(b) presents the force curve when no ssDNA was attached on the bead. Figure SI-15(c) presents the force curve when [(dT)_25_- (dC)_25_-(dA)_50_]_m_ was attached to the bead. The specific attractive force between [(dT)_25_-(dC)_25_-(dA)_50_]_m_ and the surface of Si_3_N_4_ was confirmed in Figure SI-15(c).

Figure SI-15

References:

[1] Yanagi, I., Akahori, R., Hatano, T. & Takeda, K. Fabricating nanopores with diameters of sub-1 nm to 3 nm using multilevel pulse-voltage injection. *Sci. Rep.* 4, 5000 (2014).

[2] Akahori, R. *et al*. Slowing single-stranded DNA translocation through a solid-state nanopore by decreasing the nanopore diameter. *Nanotechnology* 25, 275501 (2014).

[3] Goto, Y. *et al*. Integrated solid-state nanopore platform for nanopore fabrication via dielectric breakdown, DNA-speed deceleration and noise reduction. *Sci. Rep.* 6, 31324 (2016).

[4] Goto, Y. *et al*. Deceleration of single-stranded DNA passing through a nanopore using a nanometre-sized bead structure. *Sci. Rep.* 5, 16640 (2015).
